# How Does Servant Leadership Influences Creativity? Enhancing Employee Creativity *via* Creative Process Engagement and Knowledge Sharing

**DOI:** 10.3389/fpsyg.2022.947092

**Published:** 2022-07-01

**Authors:** Meizhao Chen, Muhammad Zada, Jawad Khan, Noor Ul Saba

**Affiliations:** ^1^School of Public Administration, Guangdong University of Foreign Studies, Guangzhou, China; ^2^Business School, Henan University, Kaifeng, China; ^3^Department of Business Administration, Alhamd Islamic University, Islamabad, Pakistan; ^4^Department of Business Administration, Iqra National University, Peshawar, Pakistan; ^5^Department of Management Sciences, Bahria University, Islamabad, Pakistan

**Keywords:** quantitative, employee creativity, servant leadership, knowledge sharing, creative process engagement

## Abstract

Grounding on social exchange theory and using the creative process engagement as a lens, this article study investigates the influence of servant leadership on employee creativity. In addition, the research examines the role of knowledge sharing in the link between servant leadership and employee creativity. Time lag method was used to collect the data from 242 employees and 57 managers employed in Chinese publicly listed companies. The data collection was divided into two parts. The subordinates were asked to rate servant leadership, their creative process engagement behavior, and knowledge sharing. The supervisors were asked to rate their associates regarding employee creativity. According to the results, employees creative process engagement behaviors mediated servant leadership and employees’ creativity. In addition, the link between servant leadership and employees’ creativity was strengthened by knowledge sharing. A detailed model is also provided, highlighting the significance of leadership, work engagement, knowledge sharing in fostering employee creativity in the Asian environment.

## Introduction

Global economic structures are being reshaped by the effects of technological changes, altering the way businesses operate, trade commodities, invest money, and create new products. The ability to think creatively and innovatively is now essential for today’s businesses to remain viable and prosper (Bauer et al., 2019; Christensen-Salem et al., 2021). Employees and managers have a crucial role in developing and maintaining creative and innovative organizational processes (Ruiz-Palomino and Zoghbi-Manrique-de-Lara, 2020). Many organizations’ competitive edge, flexibility, and long-term viability are all dependent on their employees’ and supervisors’ ability to foster a culture of creativity and new ideas (Holtzhausen and de Klerk, 2018). Therefore, understanding the role of creativity in organizations is a crucial concern for researchers in organizational behavior (Afsar et al., 2018; Wu and Zhu, 2021). Individual creative behavior at the workplace is commonly conducted in a team or group. The individual’s creative performance may contribute to the team’s creative performance and the achievement of collective goals (Allen et al., 2018). The psychological attachment with the team droves employees to participate and perform in creative endeavors. Team creativity can be enhanced by collaborative work, but individual creativity is overlooked in organizational research (Aboramadan, 2020; Iqbal et al., 2020).

Employee creativity is defined as “the generation of novel and useful ideas” (Amabile, 1988; Yu et al., 2019). There has been an upsurge in research on what leaders can do to encourage employee creativity (Imran et al., 2018; Ogbeibu et al., 2018). Leaders play a crucial role in determining the challenges faced by a team and organization and facilitate employees in developing their knowledge and skills needed for creativity accordingly. Team creativity smoothly runs while employees contribute equally and may benefit when team members have smooth knowledge exchanges. In employee development and team encouragement, servant leadership (SL) is considered the best leadership style to welcome the dual challenge of facilitating team and individual employees (Zhou et al., 2018).

*Servant leadership can be described as “a style of leadership that promotes the collective interest of employees, helping them reach collective goals* (García-Morales et al., 2012)*.”*

This research examines the influence of servant leadership on employee creativity. It also studies how servant leadership facilitates employees’ creativity *via* creative process engagement and knowledge sharing (Zada et al., 2022a). A servant leader approaches employee behavior from various perspectives to provide them with the ability to be creative and productive (Yang et al., 2019). According to the findings, an employee’s willingness to share their knowledge may help mitigate the impact of servant leadership on the creative output of the workplace. Knowledge and experience on a specific topic might lead to a person’s ability to think outside the box (Williams et al., 2017). It would be more beneficial to workers’ abilities to be creative if their leader supported open communication and the exchange of knowledge between them (Zada et al., 2022c). Employees become more creative when they share their expertise, and they have enough knowledge to come up with and put into action new ideas under the guidance of servant leadership (Su et al., 2020).

The following areas in which this research hopes to make a difference. First, the study on creative outputs seems to be extensive, but there appears to be a lack of research on employee creative process engagement (Cheng and Yang, 2019). While the study will add to the current body of knowledge on employee creativity by providing new information on how employees engage in the creative process in general, it will be instrumental in demonstrating how various components interact dynamically in different organizational settings. Second, servant leadership research in Asian cultures is relatively scarce and inconclusive (Cheng and Yang, 2019; Ali et al., 2020b). Rather than value-based leadership styles such as servant leadership, transformational, transactional leadership styles were studied in more depth (Da Costa et al., 2018; Piyathasanan et al., 2018). This research will present new empirical data and new theoretical insights on the efficiency of servant leadership in Asian hierarchical civilizations and cross-cultural management. Third, to further understand how individuals vary in their levels of creativity in the workplace, we used variables derived from the “interactionist perspective of creativity” (IPC) paradigm (Woodman et al., 1993). Drawing on the tenet of Grounding on the social exchange theory, the current study suggests that leaders’ ability to inspire their followers’ creativity and their activity relies on their relationships with their followers, as well as other context and situational elements (Li et al., 2020).

Further, this research examined employees’ creative process engagement behavior in empirical settings by combining individual and organizational characteristics. Fourth, Leaders play a vital role in building an open, trusting atmosphere, leading by example, defining standards, enabling team members to exchange ideas, and acknowledging the accomplishments of people in their team. This study explains that servant leadership focuses on organizational setting, encouraging, and executing a creative process engagement of employees; becoming a role model and ambassador focuses on aligning, caring for, and growing talent and continuously monitoring and improving employee creativity. In organizations, knowledge sharing takes the shape of entrenched culture and norms, which may help servant leaders achieve creativity among their workforce. The research outcomes will help supervisors comprehensively understand their workers’ creative behavior (Amabile and Pratt, 2016). Finally, this study will offer empirical data on employees’ creative behavior in developing countries since research on employee creativity in developing and emerging economies is scarce.

## Literature Review and Hypothesis Development

### Servant Leadership and Employee Creativity

The current era is based on innovative technologies that need top management support. Unfortunately, it is still not a vital agenda of top management. Creativity is defined as the capacity to develop something new and valuable. It is critical to the emergence of new organizations and the survival of the most successful ones after they have gone global (Neubert et al., 2008; Yang et al., 2019). Employees are motivated to collaborate when they are allowed to express themselves creatively. Collaboration is encouraged throughout the creative process. Businesses must create a constant learning attitude among their staff, pushing them to seek new information, expertise, and innovative ways of doing things (Zada et al., 2022c). According to social exchange theory (Cropanzano and Mitchell, 2005), the leader provides more benefits or regards than burden or costs to the followers who in exchange help him achieve the goals of the organization. Servant leadership style plays a significant role in employee creativity (Yoshida et al., 2014). Servant leaders are so helpful in encouraging and developing a culture of creativity among employees (Iqbal et al., 2020). Employees find new ways to perform their roles in a cooperative environment without considering failure and fears. Employees who work under servant leadership are encouraged to challenge the wrong system and try new techniques that boost employee creativity (Williams et al., 2017). According to Podsakoff et al. (2003), servant leadership comprises important characteristics that provide constant stimulation and encourage subordinates to evaluate things from a new perspective and reconsider how to do their job. Servant leadership inspires their subordinates by their visions. To ensure that everyone is working toward a single objective, you need a visionary leader who conveys their idea clearly and passionately (Tuan, 2020). Servant leadership is essential for good performance because it coordinates both the effectiveness of an employee and other resources in the organization. Servant leaders motivate employees, increase their job performance and commitment, and bring creativity to their task roles (Wang et al., 2021). Employees who work under a servant leader are more likely to explore new and better methods to do their job.

Furthermore, servant leaders help their employees to develop a more creative self-image (Van Dierendonck and Rook, 2010). Consequently, employees should feel more comfortable trying new ways to formulate creative ideas. As a result of the above arguments, we believe that servant leadership increases employees’ creativity (Ruiz-Palomino and Zoghbi-Manrique-de-Lara, 2020).

*Hypothesis 1*: Servant leadership is positively related to employee creativity.

### Servant Leadership and Employees’ Creative Process Engagement

The effects of leadership styles on the creative output of employees have been thoroughly established in previous research studies (De Sousa and Van Dierendonck, 2014). According to the social exchange theory (Blau, 1964), the reciprocal exchange connection between leaders and followers fosters employee problem identification and the search for alternatives. Various contextual and social factors influence employees’ creative behavior, including the organization’s climate. The fulfillment of tasks, rewards, and punishments, and the role models provided by managers and senior executives (i.e., creative performance or engagement in the creative process; Yuan et al., 2018). The creative ideas, one-on-one mentoring, supportive atmosphere, and intellectual stimulation abilities of servant leaders may motivate their followers to engage in creative activities and processes (Saeed et al., 2019). Employees’ motivation to engage in complicated work and creative efforts effectively increases in a supportive workplace, facilitating their pleasure and job satisfaction (Jiang and Yang, 2015; Ali et al., 2020a). In addition, this environment gives assistance and feedback in the quest for creative and optimum solutions (Zhang and Bartol, 2010). According to Hu et al. (2018), servant leadership impacts the outcomes of creative thinking in employees. Creativity employees will flourish in an atmosphere created by the leader. According to Bibi and Afsar (2018), creativity is time-consuming and risky to pursue successfully. Leaders must understand the appropriate time and method to give critical assistance to maximize followers’ creative engagement. Howell and Avolio (1993) argued that servant leaders stimulate workers’ openness, creativity, and risk-taking behavior, promoting employee creative process engagement. Based on the theoretical premises and literature evaluation discussed above, the research suggests the following hypothesis:

*Hypothesis 2*: Servant leadership positively impacts employees’ creative process engagement in organizational contexts.

### Creative Process Engagement and Employee Creativity

Problem identification, environmental scanning, data collection, solution development and assessment, and solution execution are all creative tasks that a person must engage in while responding creatively to challenges (Liden et al., 2008, 2014). This creative process “determine[s] the flexibility with which cognitive pathways are explored, the attention given to particular aspects of the task, and the extent to which a particular pathway is followed in pursuit of a solution” (Amabile, 1988). If cognitive processing is disrupted, crucial knowledge will not have been accessible or employed in problem-solving, and as a consequence, limited creativity will be the result (Kwan et al., 2018). Employees’ creativity is positively correlated with creative process engagement and the componential idea of creativity. Prior literature s previously emphasized the necessity of understanding the process that ultimately leads to innovative research solutions on employee creativity (Abdelmotaleb et al., 2018; Cheng and Yang, 2019). In empirical research, it has been shown that employees are more creative when they are actively involved in the creative process (Zhang and Bartol, 2010). Based on these results, we hypothesized that employees’ creativity would improve if they were more engaged in the creative process.

*Hypothesis 3*: Creative process engagement is positively related to employee creativity.

### The Mediating Role of Creative Process Engagement

*The Motivation-Opportunities-Ability (MOA) model hypothesizes that “employee performance can be influenced by an organization’s ability to leverage these three concepts in a win-win capacity. By win-win, both the employee and the organization benefit from efforts to apply the MOA model in the workplace* (MacInnis and Jaworski, 1989).*”*

Engagement in creative processes is a vital initial step toward creativity and precedes creative outcomes (Henker et al., 2015). The servant leadership approach, which is based on the MOA model, outlines how servant leaders influence the work results of their followers. According to the MOA model, a person’s desire to perform (motivation), the situational factors that enable their activity (opportunity), and the individual’s abilities about a particular action (capacity) are all factors that influence whether or not an act is performed (Gruen et al., 2007; Cui et al., 2020). Social exchange theory may be used to examine if and how employees are motivated to take responsibility in the context of servant leadership. Scholars have suggested that servant leadership may inspire employee creativity *via* the use of social exchanges. Servant leaders positively influence employee engagement in creative processes in many ways, and this impact depends on their motivation, opportunity, and competencies. First, employees’ intrinsic drive to contribute more to creative processes at work may be increased by servant leaders by encouraging them to do so more often. Leaders’ knowledge, skills, and abilities (KSAs) contribute to creating fair and supportive workplaces improving employees’ creativity (Eva et al., 2019; Karatepe et al., 2020). Employees who work in such supportive environments are more likely to believe that their contributions are valued and that their well-being is considered, which inspires them to try to reciprocate the organization’s generosity *via* creativity (Karatepe et al., 2019). In the course of the creative process, workers who have been given enough assistance are more likely to take the initiative and commit more time and effort to obtain information, evaluate several perspectives, and recognize issues (Cheng and Yang, 2019). Second, creativity thrives when leaders and the organization support their employees. To build a culture of innovation, leaders may help their subordinates try new things, develop, and proceed (Piyathasanan et al., 2018; Cheung et al., 2020). Employees are more engaged at work and are more likely to try new approaches to solving issues when their workplace is more open to taking risks connected to creativity (Da Costa et al., 2018; Tantawy et al., 2021). Third, the problem-solving abilities of subordinates are boosted under servant leadership. Leaders inspire and assist their followers in gaining new skills, absorbing new knowledge, and learning from one other by demonstrating openness to learning, feedback, and other people’s fresh ideas (Li et al., 2020). Based on the above arguments, we construct the following hypothesis:

*Hypothesis 4*: Creative process engagement mediates the link between servant leadership and employee creativity.

### The Moderating Role of Knowledge Sharing Behavior

According to social exchange theory, knowledge sharing reflects a “social interaction culture, involving exchanging employee knowledge, experiences, and skills through the whole department or organization” (Lin, 2007). Leadership styles and knowledge-sharing activities significantly impact employee creativity (Trong Tuan, 2017). Knowledge sharing is a collection of activities that include communicating knowledge, sharing, and helping in task-relevant thoughts, facts, and proposals amongst employees and team members (Song et al., 2015). Employees need to share their knowledge in the workplace since it fosters their creativity (Kim and Park, 2015). It is necessary for employees in an organization to continually depend on their colleagues’ knowledge acquisition (skills and experience) or to utilize explicit information already available inside the organization to perform new tasks to remain creative. The organization promotes shared knowledge practices among individuals, groups, and the organization. Likely to generate fresh ideas and concepts that may be used to establish new business prospects (Dong et al., 2017). True leaders collaborate with others to transform their expertise into projects that benefit their organizations. They set the tone for others to follow through with their behaviors and manner. Rather than becoming a knowledge bottleneck, they actively encourage and facilitate the exchange of information. Establishing a dependence on their limited talents, they attempt to educate others on being more productive and less reliant on themselves. If knowledge is power, then greatness is the mark of a leader (Gilson et al., 2013; Bengrich et al., 2020). When it comes to servant leadership, one of the most important attributes is the capacity to increase the collective motivation of subordinates (Huang et al., 2014). Subordinates’ individual and collective interests are linked together by focusing on group work and sharing beliefs or ideologies. In this way, servant leaders may contribute to the formation of a collective identity that impacts the overall effectiveness of an organization.

Furthermore, servant leaders convey departmental possibilities *via* public or frequent conversations, pushing staff to be enthusiastic and creative; as a result, collaborative performance may be enhanced (Yeh et al., 2012). They are motivated to achieve high-level creativity because their leaders have earned their confidence, loyalty, and respect (Sung and Choi, 2019). Therefore, knowledge sharing among employees is based on mutual beneficial motives, where employees share and take different ideas to facilitate each other in performing creative roles under the supervision of servant leadership.

*Hypothesis 5*: Knowledge sharing positively moderates the effect of servant leadership on employee creativity.

## Materials and Methods

### Data Collection and Pretesting

The population consisted of 242 employees and 57 departmental heads working in public listed companies in China. Information regarding all study variables, i.e., servant leadership, knowledge sharing, and creative process engagement, has been procured from employees, whereas employee creativity has been collected from supervisors. All the employees and supervisors of public listed companies have been contacted to generate the research information. At time 1, out of 332 questionnaires distributed to the employees, 267 questionnaires have been returned (Response rate = 80.42%), and out of 102 questionnaires distributed to supervisors, 86 questionnaires have been received (response rate is 84.31%). At time 2, out of 267 questionnaires, 249 were received from employees (Response Rate 93.25%), and out of 86 questionnaires, 53 were received from their supervisors. After thoroughly checking the questionnaires, some questionnaires have missing values, so we have to remove all those questionnaires. In final, 242 employees responded (97.18%), and 57 supervisors (92.98%). We may consider these responses a handsome response from both employees and supervisors. The majority of replies came from males, who accounted for 72.5% of the sample’s overall response rate. According to this metric, males control most facets of life in Chinese society. According to the findings, 62% of those who answered the survey are married. The majority of those who responded to the survey were between 30 and 35 (54%). In terms of work experience, almost 52% of individuals who responded to the survey had between 1 and 5 years of experience. Graduates made up 72% of the total sample.

We analyzed the data distribution by looking at skewness and kurtosis for each variable in the framework before doing and reviewing the regression analysis in SPSS. All values ranged from ±0.035 to ±1.798, well below the threshold values of ±0.5 and ± 2, respectively (George, 2011). This implies that our data collection has a normal distribution. We also looked at the reliability and validity of the measuring model used in this research. Loading of all items ranging between 0.74 and 0.86 (see Table 1). Composite reliability ratings are well above the threshold value of 0.7, indicating high reliability. The average variance extracted (AVE) from the measurement model may also be used to assess the validity. The AVEs were in the range of 0.62–0.64, which is much higher than the criterion of 0.5. To establish discriminant validity, we additionally evaluate the AVE square root. This study’s AVE square root values for the latent variables were significantly higher than their correlations with the other latent variables, indicating strong discriminant validity (see Table 1). The data for the study variables have been obtained from a single source, leading to the prevalent bias issue (Podsakoff et al., 2003). This issue was investigated using Harman’s -factor test (Lindell and Whitney, 2001). The results indicated that the variation explained by a single component is about 25.42% of the total variance. Overall, the information shown in the preceding figures suggests that the measurement model is reliable and valid. Consequently, it may be concluded that all structures are suitable for future investigation.

**Table 1:** Factor loadings.

Constructs	Items	Factor Loadings	CR	AVE	√AVE
**Servant leadership**	**SL1**	**0.83**	**0.92**	**0.62**	**0.78**
	SL2	0.77			
	SL3	0.81			
	SL4	0.77			
	SL5	0.77			
	SL6	0.75			
	SL7	0.82			
**Knowledge sharing**	**KS1**	**0.81**	**0.83**	**0.62**	**0.79**
	KS2	0.78			
	KS3	0.79			
**Creative process engagement**	**CPE1**	**0.78**	**0.95**	**0.64**	**0.79**
	**CPE2**	**0.86**			
	CPE3	0.84			
	CPE4	0.83			
	CPE5	0.74			
	CPE6	0.71			
	CPE7	0.79			
	CPE8	0.92			
	CPE9	0.76			
	CPE10	0.78			
	CPE11	0.81			
**Employee creativity**	**EC1**	**0.81**	**0.86**	**0.62**	**0.78**
	EC2	0.77			
	EC3	0.82			
	EC4	0.76			

### Measures

#### Servant Leadership

The servant leadership was measured by Liden et al. (2015) seven-item scale. One sample item is “My leader can tell if something work-related is going wrong.” Cronbach’s α coefficient for this scale is 0.93.

#### Creative Process Engagement

Creative process engagement was assessed using an 11-items scale (α = 0.92) by Zhang and Bartol (2010). For example, “I spend a lot of time looking for the essence of the problem.”

#### Employee Creativity

Employee creativity was assessed using a 4-items scale developed by Farmer et al. (2003). For example, “This employee: Tries new ideas or methods first.”

#### Knowledge Sharing

Knowledge Sharing was assessed using 3-items scale developed by Choi et al. (2010). Sample items are, “Our team members share their experience or know-how from work with other team members.”

#### Control Variables

According to previous study, employee demographic characteristics may account for variation in their creativity, which might alter the outcomes of the hypothesized correlations (George and Zhou, 2001; Shin and Zhou, 2003). As a result, we used age, gender, education level, and tenure with the company as control variables.

#### Descriptive Statistics and Correlation

Table 2 shows the research variables’ mean values, standard deviations, and correlations. The zero-order correlations for servant leadership, employee creativity, knowledge sharing, and creative process engagement were all in the anticipated direction, consistent with our theoretical assumptions. Servant leadership and employee creativity have the most significant link (*r* = 0.547, *p* < 0.01). See Table 2 for further details.

**Table 2:** Mean, SD, correlations, and reliability.

**S. No.**	**Variables**	**Mean**	**SD**	**1**	**2**	**3**	**4**	**5**	**6**	**7**
1.	Age	2.43	0.82							
2.	Education	2.86	0.58	0.041						
3.	Experience	1.99	0.70	0.051	−0.034					
4.	Servant Leadership	3.88	0.77	0.075	0.053	0.048	**(0.84)**			
5.	Creative Process Engagement	3.81	0.62	0.040	0.078	−0.025	0.467^**^	**(0.75)**		
6.	Employee Creativity	4.12	0.79	0.105	0.107	0.006	0.547^**^	0.535^**^	**(0.81)**	
7.	Knowledge Sharing	3.57	0.77	0.087	0.215^**^	0.020	0.364^**^	0.406^**^	0.508^**^	**(0.77)**

** Correlation is significant at the 0.01 level (2-tailed).

#### Confirmatory Factor Analysis

We conducted multi-level confirmatory factor analyses to verify the discriminability of the measures. We incorporated study variables servant leadership (SL), employee creativity (EC), creative process engagement (CPE), and employee creativity (EC) to check model structure fitness. Table 3 shows that the one-factor model best fits the data compared to other models (*X*^2^ = 2,374, df = 1,261, TLI = 0.91, CFI = 0.92, RMSEA = 0.03, SRMR = 0.04).

**Table 3:** Results of the confirmatory factor analysis (*N* = 299).

Model’s	*X* ^2^	*df*	TLI	CFI	RMSEA	SRMR
Hypothesized one-factor model	2,374	1,261	0.91	0.92	0.03	0.04
Two-factor model:	3,356	3,247	0.53	0.63	0.15	0.11
Three-factor model:	5,360	4,256	0.42	0.54	0.17	0.15
Four-factor model: SL, EC, KS and CEP	6,374	5,227	0.32	0.42	0.23	0.26

*X*^2^ = normal-theory weighted least-squares Chi-square. TLI, Tucker–Lewis fit index; CFI, Comparative fit index; RMSEA, Root-mean-square error of approximation; SRMR, Standardized root-mean-square residual.

#### Direct Paths and Mediation Effect

Table 4 shows the direct relationship between HI, H2, and H3. Results illustrate that SL positively and significantly affects employee creativity (*β* = 0.639, *p* < 0.001), supporting first hypothesis of the study (H1). Hypotheses 2 (H2) suggest that a positive link exists between SL and creative process engagement such that (*β* = 0.680, *p* < 0.001), confirming the second hypotheses. We further tested for hypotheses 3. Table 4 shows that the creative engagement process positively affects employee creativity (*β* = 0.873, *p* < 0.001), endorsing hypotheses 3 (H3). The study model tested for mediation (see Table 5), shows that employee creative process engagement partially mediates that relation between SL and employee creativity such that [*β* = 0.4719, Boot SE = 0.0611 and (Boot LLCI = 0.3535 Boot ULCI = 0.5918)]. Zero is not located between confidence intervals at 95%, supporting our hypothesis four (H4).

**Table 4:** Path analysis (direct relationship).

Hypotheses	*R* ^2^	*B*	*t*-Test	*p*	Decision
H1	0.38	0.639	13.72	0.000	38% variation in EC due to SL
H2	0.70	0.680	27.08	0.000	70% variation in CPE due to SL
H3	0.46	0.873	16.64	0.000	46% variation in EC due to CPE

Hypotheses tested at a confidence interval of 95%.

**Table 5:** Mediation analysis.

	***β***	**SE**	*t*	*p*	**95%CI**	
					LL	UL
**Step 1**						
Constant	0.8060	0.2018	3.9943	0.0001	0.4090	1.2031
SL	0.1503	0.0776	1.9362	0.0537	−0.0024	0.3030
EC	0.7184	0.0956	7.5184	0.0000	0.5304	0.9064
	***β***	**BootSE**	**BootLLCI**	**BootULCI**	**Decision**	
**Step 2**						
Mediation path	0.4719	0.0611	0.3535	0.5918	Partial Mediation	

#### Moderation Effect

**Figure 1:**
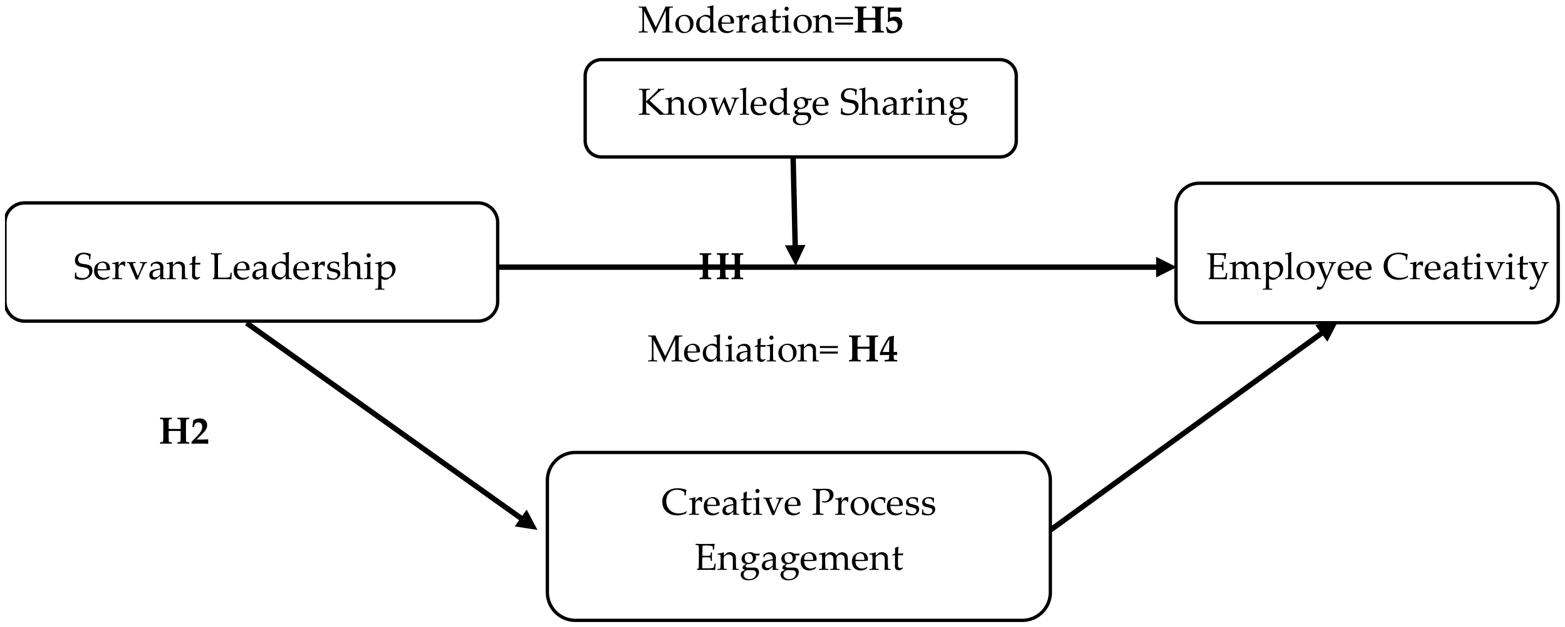
Conceptual model.

**Figure 2:**
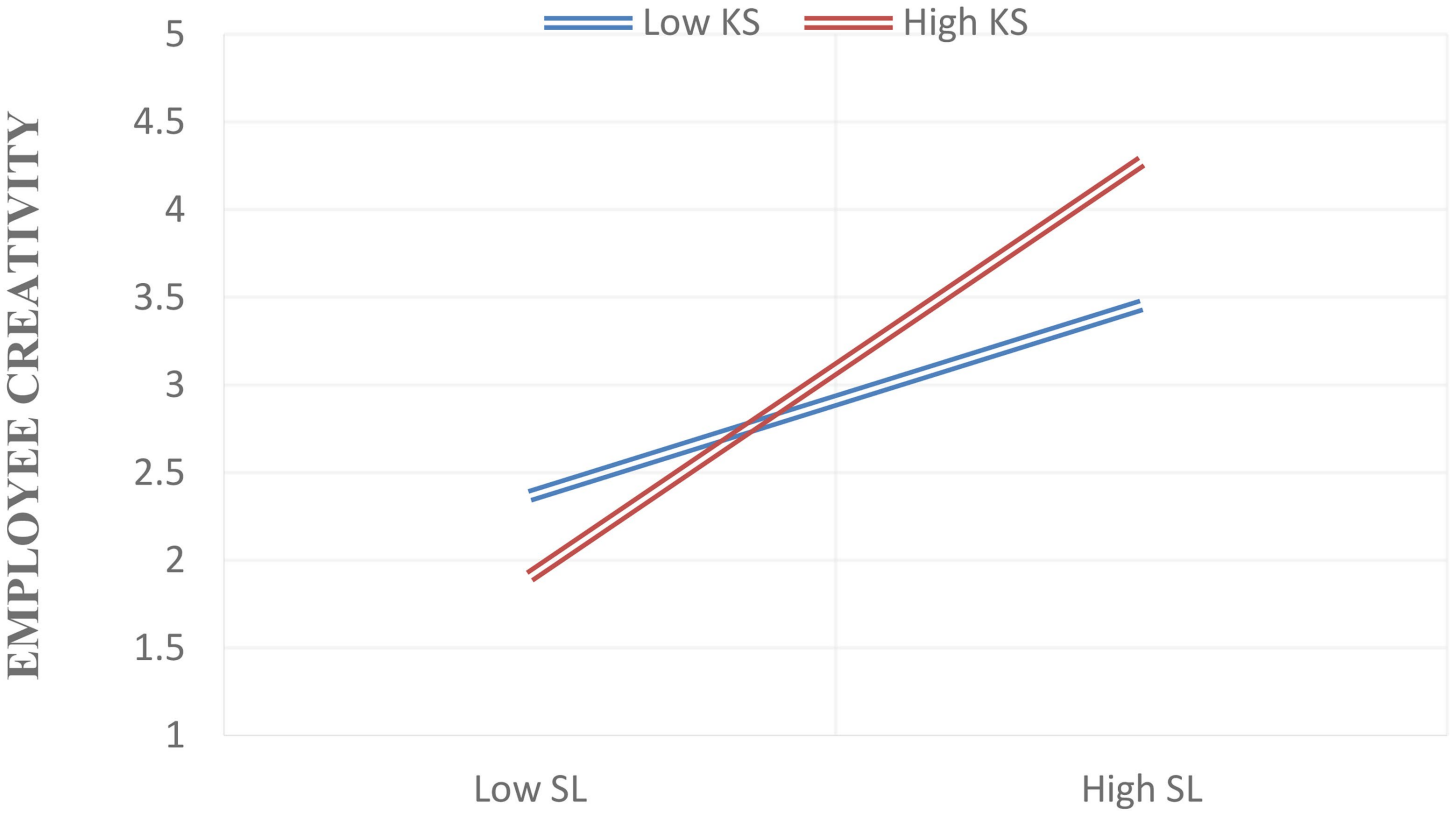
Interaction model.

Hayes (2017) Process Macro Model 1 has been applied to test the moderation. Table 6 and Figure 1, shows the moderating effects of knowledge sharing between SL and employee creativity (*b* = −0.1122, SE = 0472, *t* = −2.3765, *p* = 0.0181, [LLCI = −0.2050 ULCI = −0.0193]), supporting fifth Hypotheses (H5), such that a knowledge sharing strengthens the relationship. To make the moderating impact of knowledge sharing more visible, this research computed two kinds of knowledge sharing mean: one with a standard deviation and the other with a lower standard deviation, as suggested by Aiken et al. (1991). Figure 2 depicts the interactive mode.

**Table 6:** Moderation analysis.

Variables	SL (X)		Knowledge sharing (W)		EC(Y)		
	*β*	SE	*t*	*p*	95%CI		
					LL	UL	
Constant	−0.7332	0.6188	−1.1847	0.2370	−1.9508	0.4845	
SL	0.8636	0.1699	5.0829	0.0000	0.5293	1.1979	
KS	0.8436	0.1780	4.7390	0.0000	0.5134	1.2138	
SL ^*^ KS	−0.1122	0.0472	−2.3765	0.0181	−0.2050	−0.0193	
**HP (−1 SD)**	0.5501	0.0532	10.3433	0.0000	0.4455	0.6548	
**HP (−1 SD)**	0.3751	0.0586	6.4072	0.0000	0.2599	0.4904	
**R** ^2^							**0.55** ^***^
**ΔR** ^2^							**0.0080** ^*^

* *p* < 0.01;

*** *p* < 0.001.

Moderator values are the mean and ± 1 SD, LLCI, lower limit 95% confidence interval; ULCI, upper limit 95% confidence interval.

## General Discussion

Servant leadership was shown to increase employee creativity, and these findings were in accordance with earlier research, although limited research has been done to establish between servant leadership and employee creativity (Liden et al., 2014; Williams et al., 2017). Employees are more likely to feel at ease and exhibit trust in their leaders when they perceive them to be cautious and less self-centered, which in turn helps them increase problem-solving efforts and employee creativity (Yoshida et al., 2014). These findings lend support to the theoretical arguments that are based on the social exchange theory. According to this theory, if there are quality social exchanges between the leader and the followers in the workplace, then the followers are more likely to reciprocate the positive behaviors by engaging in creative behavior. As a result, when followers have a good view of their servant leaders, they are more likely to show their gratitude in creative ways (Cropanzano and Mitchell, 2005). Servant leadership was shown to have a positive effect on employee creative process engagement. According to prior study, the presence of leadership has a considerable impact on participation in creativity-relevant procedures or processes because leaders play an important role in creating a creative environment inside the organization (Reiter-Palmon and Illies, 2004; Siyal et al., 2021).

Previous research has shown that servant leadership has a good influence on staff creativity. However, there has only been a limited amount of study done on the workers of publicly listed companies (Eva et al., 2019; Khan et al., 2022b). Servant leaders believe that it is their responsibility to assist others in learning and growing, feeling meaningful, inspired, energized, and contributing at their maximum level. People are encouraged to conduct good work that brings out the best in them when they are under the direction of servant leadership. Instead of teaching them how to do a better job, you are more likely to engage them by asking them how you can assist them in doing their jobs better. You put the needs of your people ahead of your interests. If, on the other hand, you regard your people as a method of gaining glory, fortune, and recognition for your organization or your leadership, you are most likely not a servant leader in this sense.

Additionally, the results of this study have implications for cross-cultural management and leadership studies since they provide fresh empirical evidence on the effectiveness of servant leadership in Asian hierarchical cultures. Succeeding studies show a link between servant leadership and creative process engagement *via* increased work difficulties and issues, supporting the idea that servant leaders play a significant role in encouraging others to take the initiative (Gandolfi and Stone, 2018; Pawar et al., 2020). Employees’ opinions and experiences impact their leaders’ ability to solve problems throughout their workday, as shown by the substantial positive correlation between servant leadership and creative process engagement approaches (Lee et al., 2020).

Employees who work under a servant leader are more likely to take the initiative and seek out social connections, expertise, and feedback to improve their day-to-day tasks (Ali et al., 2021; Ullah et al., 2021). In addition, servant leaders may help create a dynamic learning environment in their workplaces (Lee et al., 2020). They prepare their followers to participate in the creative process and advance in their professional careers due to their actions. The link between leadership role and proactive behavior at work has been examined in the literature but less attention has been given to examine the leadership role in employee creative process engagement. The findings of this study shows that servant leadership and employee creative process engagement are positively linked and also indicate that leader’s play important role in the creative process engagement process (Andersen, 2018). The relationship between servant leadership and employee proactivity is widely proven, yet there is always room for improvement. According to research, this is the first study to demonstrate that employee engagement in the creative process may mediate the relationship between servant leadership and employee creativity.

Finally, when it comes to servant leadership and employee creativity, we examined the role of knowledge sharing as a buffering factor. Instead of focusing on explicit knowledge and tacit knowledge, as earlier studies did, our research verified knowledge sharing as a reflective notion that can be evaluated by knowledge transfer. This was done to ensure compatibility with the study purpose, which posits that knowledge sharing and receiving would engage workers into employee creativity while working under a servant leader (Carter and Baghurst, 2014; Hughes et al., 2018). This would imply that knowledge sharing might be an enabling condition for servant leadership to perform effectively. Also noteworthy is that managers may utilize knowledge sharing to encourage their staff to be more creative in the workplace.

### Practical Implications

Organizations, particularly those in the corporate sector looking for quick and efficient solutions to a competitive market climate, should put an emphasis on encouraging their workers’ creativity. The most important prerequisite for management is to provide a supportive creative working environment in which employees feel empowered and get customized attention, incentives, and acknowledgment for their creative efforts. Servant leadership may be utilized to establish a creative atmosphere and hence boost the involvement of followers in creative activities. The selection, training, and development of servant leaders is advocated for managers in order to promote and create an environment conducive to creative activity. This is why training programmes for servant leaders that enhance their abilities to promote an environment of creativity may be used to increase their effectiveness. A manager’s responsibility is to help his or her subordinates think outside the box and come up with new and unique solutions and ideas.

Given the significance of employee creativity for organizational performance (Swanson et al., 2020), leaders need to understand how they can encourage the creative thinking of their people in the workplace. Leaders should demonstrate servant leadership traits, according to our findings. Servant leaders encourage employees to share their thoughts, vision and ideas with their colleagues to foster creativity in the organization. Considering that leadership style is one of many antecedents to encourage employees’ creativity (Weber et al., 2018). Organizations must create a favorable setting for creative process engagement practices while also limiting the possibility of creative limitations occurring in the organization setup. Instead of preventing failure, servant leadership should create a culture that welcomes success. Furthermore, our findings show the significance of employee engagement in the creative process for fostering individual creativity. Rather than directly examining the link between servant leadership and employee creativity, we looked at how employees’ engagement in the creative process influences this relationship. In order to encourage workers’ participation in the creative process, managers need to set aside sufficient amounts of time for appropriate problem identification, encourage workers’ knowledge search and encoding by providing resources, and let workers to generate ideas (Chen and Lin, 2018).

### Theoretical Contribution

Theoretically, in response to the calls from the academic community to enhance the corpus of knowledge about servant leadership, which is still in its developmental stage (Eva et al., 2019). Even more importantly (Karatepe et al., 2019; Zada et al., 2022b) called for the need to further explore the mechanism by which servant leadership influences individual and organizational results. This is a very important call to action. These results show how servant leadership promotes creativity by introducing an intervening mechanism: creative process engagement. Second, this study examines the effect of servant leadership on creativity in publicly listed organizations, where little is known about how servant leaders’ actions influence workers’ behaviors (Ling et al., 2017; Zada et al., 2022c).

Establishing a supportive work atmosphere where employees feel valued and respected is a significant feature of servant leadership. Servant leadership can help to create a more constructive professional atmosphere with a high level of enthusiasm and dedication. Businesses may develop, and workers can feel empowered if they show compassion, empathy, humility, and service. This, in turn, allows for more significant firm development (Khan et al., 2022a). A novel model of servant leadership that promotes employee creativity *via* the mediation of creative process engagements is developed and tested in this research, which makes a significant contribution to the servant leadership literature in the Asian context (China). Even though servant leadership has proven to influence employee outcomes, other aspects of the process are currently examined in this study (Lemoine et al., 2019). Our research examined further by demonstrating the importance of the creative process engagement as a linking mechanism for the servant leadership creativity.

Additionally, our research extended past research in this area by studying creative process engagement as a mechanism that mediates the link between servant leadership and employee creativity. The findings are consistent with the hypothesis that servant leaders encourage their staff to participate in the creative process to bring creativity in their respective roles (Gandolfi et al., 2017; Saeed et al., 2022). The results of this study, which investigated the impact of servant leadership on employees’ creative process engagement, could contribute to the advancement of knowledge about the interaction effects of creative behavior on employees’ creativity. As previously stated, there has been no conclusive evidence of the efficacy of servant leadership in Asian cultures until recently. The outcomes of this study will contribute to the body of research about the effectiveness of servant leadership in Asian hierarchical systems, which is currently lacking. Employees may benefit from their coworkers’ expertise by exchanging ideas and learning from one another’s perspectives *via* knowledge sharing. Knowledge sharing in an organization increases the likelihood that workers will make the correct choice and develop the best solution. Lastly, this study fills a research gap on employee creativity in developing and emerging economies by providing verifiable data on this topic (Heyler and Martin, 2018; Khan et al., 2021).

## Conclusion

To inspire and assist their followers, servant leaders may empower their followers, prioritizing their needs, and igniting their maximum potential. As a result, workers’ intrinsic drive and participation in creative activities increase. Employees are more likely to participate in creative collaboration, share knowledge and assist colleagues when their leaders demonstrate servant leadership qualities. Servant leaders have the power to increase the intrinsic motivation and the level of cognitive risk-taking of employees. Servant leaders share their expertise and inspire their staff to engage in creative process to foster an individual’s creativity. Providing intellectual stimulation encourages people to think outside the box and develop creative ideas.

## Data Availability Statement

The original contributions presented in the study are included in the article/supplementary material, further inquiries can be directed to the corresponding author.

## Author Contributions

MC and MZ contributed to the conception and design of the study. JK organized the database. JK and NS performed the statistical analysis. MC, MZ, and JK wrote the first draft of the manuscript. NS wrote sections of the manuscript. All authors contributed to manuscript revision, read, and approved the submitted version.

## Funding

This work was sponsored in part by General Program of humanities and Social Sciences Research, Ministry of Education, China (19YJA630009).

## Conflict of Interest

The authors declare that the research was conducted in the absence of any commercial or financial relationships that could be construed as a potential conflict of interest.

## Publisher’s Note

All claims expressed in this article are solely those of the authors and do not necessarily represent those of their affiliated organizations, or those of the publisher, the editors and the reviewers. Any product that may be evaluated in this article, or claim that may be made by its manufacturer, is not guaranteed or endorsed by the publisher.
